# BioMNEDR: mechanism-guided network embedding for drug repurposing

**DOI:** 10.1093/bib/bbag101

**Published:** 2026-03-09

**Authors:** Yizhou Zeng, Lei Wang, Xueming Liu

**Affiliations:** School of Future Technology, Huazhong University of Science and Technology, Luoyu Road, 430074 Wuhan, China; School of Artificial Intelligence and Automation, State Key Laboratory of Digital Manufacturing Equipments and Technology, Institute of Medical Equipment Science and Engineering, Luoyu Road, 430074 Wuhan, China; School of Artificial Intelligence and Automation, State Key Laboratory of Digital Manufacturing Equipments and Technology, Institute of Medical Equipment Science and Engineering, Luoyu Road, 430074 Wuhan, China

**Keywords:** drug repurposing, heterogeneous network, meta-path, multi-scale mechanisms

## Abstract

Drug repurposing provides a cost-effective and time-efficient strategy to accelerate therapeutic discovery, yet most computational approaches fail to capture the multi-scale biomedical mechanisms underlying drug–disease associations, limiting interpretability. We introduce BioMNEDR (mechanism-guided network embedding for drug repurposing) that integrates heterogeneous biomedical networks through biologically curated meta-paths. BioMNEDR generates low-dimensional embeddings preserving protein–protein interactions and functional hierarchies. It further integrates multi-path predictions through an XGBoost classifier. The framework achieves state-of-the-art performance, consistently surpassing strong baselines across AUROC, AUPR, recall, and F1-score, while maintaining a balanced trade-off in precision. Case studies further highlight its practical utility, demonstrating the ability to rediscover approved drugs and prioritize promising candidates, such as cromoglicic acid for Alzheimer’s disease. By explicitly modeling multi-scale mechanisms, BioMNEDR enhances both predictive accuracy and biomedical interpretability, offering a robust computational framework for systematic drug repurposing.

## Introduction

Drug repurposing—the strategy of identifying new therapeutic uses for approved or investigational compounds—has emerged as an urgent alternative to *de novo* drug development, given the escalating costs, prolonged timelines, and declining success rates of traditional pipelines [1]. Historically, most successful cases have arisen from serendipitous clinical observations. However, with the exponential growth of biomedical data, drug repurposing has evolved from anecdotal discoveries to a systematic, data-driven discipline, supported by computational modeling [2].

A variety of computational strategies have been explored, including molecular docking [3, 4], clinical data mining [5], and signature matching approaches, such as the Connectivity Map [6, 7]. While effective in specific contexts, these methods often rely on localized molecular features and fail to capture the multi-scale mechanisms that shape drug–disease relationships. As most diseases emerge from disruptions in coordinated biological processes rather than isolated single-gene defects, advancing repurposing requires models that move beyond single-entity associations to embrace systems-level interactions, as exemplified by frameworks that integrate biologically grounded graph construction for robust cell type annotation [8].

Network-based approaches have proven powerful in integrating heterogeneous biomedical knowledge and uncovering latent drug–disease associations. Representative examples include diffusion algorithms [9], proximity-based measures [10], AI-driven models such as graph convolutional networks [11], prototype-guided frameworks that emphasize interpretable embeddings through gene interaction mining [12], and consensus multi-graph learning approaches that leverage multiple mechanism-level graphs to enhance robustness and interpretability [13]. To reflect the latest advancements in graph-based biomedical prediction, state-of-the-art models like the Personalized Propagation Auto-Encoder model for predicting Drug-Target Interactions (PPAEDTI) [14] specialize in drug–target interaction prediction with rigorous benchmarking, including network-aware negative sampling and multiple evaluation splits. Despite their predictive capacity, these approaches remain largely opaque, offering limited mechanistic insights into their outputs. This lack of interpretability undermines confidence in their outputs and constrains their translation into clinical and regulatory practice.

Here, we present BioMNEDR, a mechanism-guided network embedding framework for drug repurposing. The main contributions are as follows:

- **Mechanism-driven design**: We advance drug repurposing by curating biologically meaningful meta-paths that explicitly capture multi-scale drug mechanisms, enhancing interpretability beyond black-box embeddings.
- **Robust embedding and prediction**: We enable comprehensive modeling of polypharmacological effects through a meta-path-based embedding method combined with XGBoost and a multi-path integration strategy.
- **Superior performance and clinical relevance**: We validate BioMNEDR against state-of-the-art baselines across multiple evaluation metrics and demonstrate its ability to recover and prioritize clinically relevant candidates in Alzheimer’s disease (AD), Parkinson’s disease (PD), and breast cancer (BC).

## Materials and methods

### Multiscale interactome network

We employed an MSI network [15] that is a heterogeneous network designed to clarify drug therapeutic mechanisms by integrating protein–protein interactions and hierarchical biological functional relationships. The MSI network contains four node types (drugs, diseases, proteins, and biological functions) and five edge types (drug–protein, disease–protein, protein–protein, protein–function, and function–function). While recent biomedical knowledge graphs have expanded in scale, MSI was specifically selected for its unique integration of hierarchical biological functions alongside molecular interactions. Unlike standard heterogeneous networks that rely primarily on entity-level associations, MSI explicitly characterizes the functional landscape through protein–function and function–function hierarchies. This integrated structure enables systematic modeling of both physical interactions and functional regulatory effects, thereby providing a biologically interpretable foundation for drug repurposing. Detailed node and edge statistics are summarized in Tables 1 and 2.

**Table 1:** Node statistics of the MSI network [15].

Node type	Number
Drugs	1661
Diseases	840
Proteins	17 660
Biological function	9798

**Table 2:** Edge statistics of the MSI network [15].

Edge type	Number
drug–protein [16–18]	8568
disease–protein [19]	25 212
protein–protein [16, 20, 21]	387 626
protein–biological function [22–24]	34 777
biological function to biological function [23, 24]	22 545

### Overview of BioMNEDR

**Figure 1:**
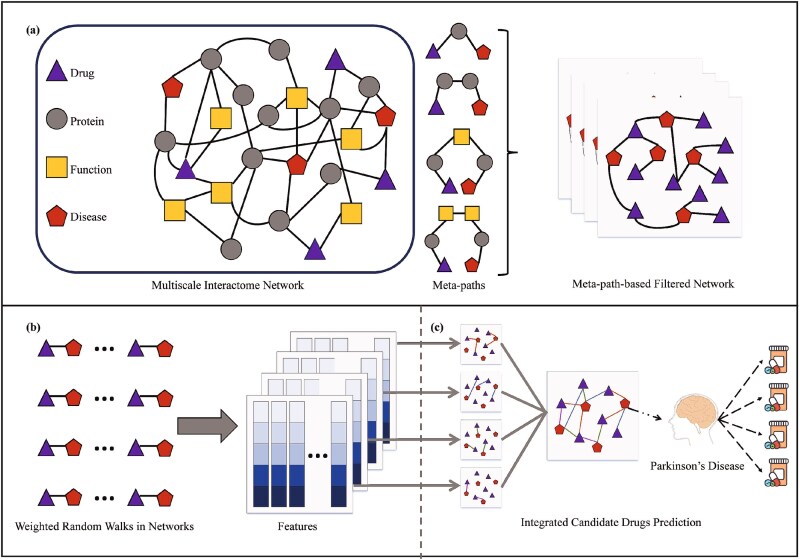
The workflow of BioMNEDR. (a) Biomedical mechanism-driven meta-paths are defined for the MSI network to model multi-level drug actions. Weighted drug–disease networks are constructed under each meta-path, quantifying associations mediated by proteins and biological functions. (b) Edge weight-guided random walks are performed on the filtered networks, and the resulting walk sequences are incorporated into a heterogeneous skip-gram model to learn embeddings that preserve topological and semantic dependencies. (c) XGBoost classifiers are trained on the embeddings derived from each meta-path, and predictions are integrated using a MAX strategy, whereby a drug is identified as a candidate for a given disease if any meta-path model predicts their association.

BioMNEDR comprises three core components: (i) construction of meta-path-based filtered networks for drug–disease associations, (ii) heterogeneous network embedding learning, and (iii) XGBoost-based prediction with final score integration. These steps collectively enable BioMNEDR to capture multi-scale biomedical mechanisms and generate interpretable predictions for drug repurposing. An overview of the workflow is presented in Fig. 1.

### Meta-path-based network construction

We manually define meta-paths based on established biomedical mechanisms to capture multi-level drug actions, including direct targeting, functional regulation, and cascade effects [10, 25]. The selection of meta-paths was guided by two core principles: (i) preserving biological interpretability by aligning with known drug–disease interaction mechanisms and (ii) limiting path length to maintain therapeutic relevance. Specifically, overly long paths (exceeding five nodes) were excluded based on empirical and methodological literature indicating that such extended paths tend to introduce semantic noise and weaken mechanism-specific associations [26]. The final meta-paths include $\mathrm{Dr{-}P{-}Di}$, $\mathrm{Dr{-}P{-}P{-}Di}$, $\mathrm{Dr{-}P{-}F{-}P{-}Di}$, and $\mathrm{Dr{-}P{-}F{-}F{-}P{-}Di}$, where $\mathrm{Dr}$, $\mathrm{P}$, $\mathrm{F}$, and $\mathrm{Di}$ denote drugs, proteins, biological functions, and diseases, respectively. These choices are supported by prior biological knowledge and validated through comparative experiments (Table 3). The chosen configuration exhibited the best overall performance, achieving the highest AUROC (0.873), AUPR (0.889), and recall (0.893) among all evaluated alternative sets. Together, these results demonstrate that the selected meta-paths successfully balance biological interpretability with robust predictive capability.

**Table 3:** Comparison of results across different meta-paths combinations. (A) $\mathrm{Dr-P-Di}$, $\mathrm{Dr-P-P-Di}$, $\mathrm{Dr-P-F-P-Di}$, and $\mathrm{Dr-P-F-F-P-Di}$; (B) $\mathrm{Dr-P-Di}$, $\mathrm{Dr-P-P-Di}$, and $\mathrm{Dr-P-F-P-Di}$; (C) $\mathrm{Dr-P-Di}$, $\mathrm{Dr-P-P-Di}$, and $\mathrm{Dr-P-F-F-P-Di}$; (D) $\mathrm{Dr-P-Di}$, $\mathrm{Dr-P-P-Di}$, $\mathrm{Dr-P-P-P-Di}$, and $\mathrm{Dr-P-F-P-Di}$; (E) $\mathrm{Dr-P-Di}$, $\mathrm{Dr-P-P-Di}$, and $\mathrm{Dr-P-P-P-Di}$. It is important to note that combination A was the primary meta-paths configuration utilized throughout the experiments described in this manuscript.

	A	B	C	D	E
AUROC	**0.873**	0.868	0.867	0.859	0.859
AUPR	**0.889**	0.880	0.879	0.872	0.873
Accuracy	0.758	**0.770**	**0.770**	0.750	0.761
Precision	0.711	**0.730**	0.726	0.702	0.719
Recall	**0.893**	0.855	0.867	0.871	0.857
F1	**0.792**	0.788	0.791	0.777	0.782

In large-scale networks such as the MSI, data sparsity and missing associations often lead to meta-path inaccessibility, limiting the effectiveness of conventional random-walk-based methods [27]. To address this issue, BioMNEDR extracts drug–disease subnetworks corresponding to the predefined meta-paths from the MSI. Formally, we denote the MSI as $G = (V, E, T_{V}, T_{E})$, where $V$ and $E$ are the sets of nodes and edges, and $T_{V}$, $T_{E}$ are the sets of node and edge types (Tables 1 and 2). For a given meta-path 


\begin{align*} & P: T_{1} \xrightarrow{R_{1}} T_{2} \xrightarrow{R_{2}} \cdots \xrightarrow{R_{k-1}} T_{k}, \end{align*}

the adjacency matrix of the corresponding meta-path-based filtered network is defined as 


\begin{align*} & A_{P} = \prod_{i=1}^{k-1} A_{R_{i}}, \end{align*}

where $A_{R_{i}}$ denotes the adjacency matrix for edge type $R_{i}$. The element $A_{P}(i,j)$ quantifies the semantic association strength between nodes $i$ and $j$ under meta-path $P$, by counting the number of distinct paths connecting them.

For example, the adjacency matrix of the $\mathrm{Dr{-}P{-}Di}$ network is given by 


\begin{align*} & A_{\mathrm{Dr{-}P{-}Di}} = A_{\mathrm{Dr{-}P}} \cdot A_{\mathrm{P{-}Di}}, \end{align*}

where $A_{\mathrm{Dr{-}P{-}Di}}(i,k)$ measures the strength of association between drug $i$ and disease $k$ mediated by shared proteins. In this way, meta-path-based drug–disease networks capture specific biological mechanisms underlying therapeutic associations.

### Heterogeneous network embedding

After constructing the meta-path-based filtered networks, BioMNEDR converts their adjacency matrices into probability distributions to enable random walks. Given the filtered network adjacency matrix $A_{P}$ under meta-path $P$, we row-normalize the transition probabilities for each node $v_{i}$ as 


\begin{align*} & P(v_{j} \mid v_{i}) = \frac{A_{P}(i,j)}{\sum_{k \in \mathcal{N}(v_{i})} A_{P}(i,k)}, \quad \forall v_{j} \in \mathcal{N}(v_{i}), \end{align*}

where $\mathcal{N}(v_{i})$ denotes the set of neighbors of $v_{i}$ in the filtered network.

Starting from the set of drug and disease nodes $S = \{v \in V \mid \phi (v) \in \{\mathrm{Dr}, \mathrm{Di}\}\}$, we generate $N$ random walks of length $L$ according to these transition probabilities. This process yields a set of weighted random walk sequences guided by meta-path $P$.

Finally, the embeddings of drugs and diseases are learned through a heterogeneous skip-gram model [28], which captures both topological and semantic dependencies across multi-hop meta-paths. These embeddings preserve multi-scale biological semantics and serve as feature inputs for subsequent prediction.

### Prediction and integration with XGBoost

Following the generation of drug and disease embeddings, an XGBoost classifier [29] is employed to predict drug–disease associations. Each input vector is obtained by concatenating the embedding of a drug with that of a disease, and the classifier is trained as a binary classification task. Known drug–disease associations are labeled as positive samples, while negative samples are constructed by randomly pairing drugs and diseases not known to be associated. To mitigate class imbalance, for each positive example two negative examples are generated: one by randomly selecting an unrelated drug and one by selecting an unrelated disease.

To integrate predictive information from multiple biomedical mechanisms, BioMNEDR adopts a MAX strategy across meta-paths. Each component model outputs a continuous probability score $y^{P_{i}}_{\mathrm{pred}}\in [0,1]$, representing the likelihood of an association from that specific biological perspective. The final prediction score is defined as 


\begin{align*} & y^{\mathrm{final}}_{\mathrm{pred}} = \max \big( y^{P_{1}}_{\mathrm{pred}}, y^{P_{2}}_{\mathrm{pred}}, y^{P_{3}}_{\mathrm{pred}}, y^{P_{4}}_{\mathrm{pred}} \big). \end{align*}

For association classification, a fixed decision threshold of 0.5 is applied to this final score to identify positive associations, enabling the calculation of standard metrics such as Accuracy, Precision, and Recall. In addition, a threshold-independent evaluation is conducted by calculating the AUROC and AUPR curves, which provides a comprehensive assessment of the model’s overall ranking and discriminative ability across all possible thresholds. This integration strategy ensures comprehensive coverage, identifying a pair as a potential candidate if it is strongly supported by at least one mechanism-specific path.

## Results

### Performance benchmarking against state-of-the-art models

To evaluate the performance of BioMNEDR, we adopted the MSI dataset curated by Ruiz *et al.* [15] that contains 5926 validated drug–disease pairs. Five-fold cross-validation was performed, and BioMNEDR was compared against several state-of-the-art methods:

- **MSI-LR** extracts diffusion-based embeddings $V_{d}$ (drug) and $V_{i}$ (disease) from the pretrained MSI model [15], concatenates them into $X(d,i)=[V_{d}\oplus V_{i}]$, and trains a logistic regression classifier on concatenated drug–disease feature vectors on these combined vectors to predict drug–disease associations.
- **DRGCC** [30] combines drug structures, disease symptoms, and gene networks through GraphSAGE with clustering constraints, using matrix factorization to predict drug–disease associations.
- **LaGAT** [31] is a link-based graph attention model for DDI prediction, dynamically constructing attention pathways using the embedding of a drug to prioritize relevant neighbors.
- **TAGCN** [32] employs typed attention to integrate entity features and relation-aware neighborhood information, refining cross-lingual entity alignment through adaptive graph aggregation.
- **GCMM** [33] fuses multimodal drug–disease similarities in a heterogeneous network, using a GCN-based attention mechanism to enhance association prediction.

As shown in Table 4, BioMNEDR consistently achieves superior results across AUROC, AUPR, recall, and F1-score, establishing new state-of-the-art performance. Notably, BioMNEDR achieves a 3.8% improvement in AUPR compared with the second-best model (LaGAT). In terms of recall, BioMNEDR outperforms the second-ranked model GCMM by 4.7%, highlighting its ability to capture more potential therapeutic associations. AUPR is recognized as the core evaluation metric for drug repurposing, as it robustly quantifies the trade-off between precision and recall. Recall is also important in repurposing, as missing viable candidates may incur substantial opportunity costs, especially at the candidate-screening stage [34].

**Table 4:** Performance comparison between baseline models and BioMNEDR on the MSI network.

Metric	DRGCC	LaGAT	TAGCN	GCMM	MSI-LR	BioMNEDR
AUROC	0.814	0.837	0.789	0.775	0.817	**0.873**
AUPR	0.816	0.841	0.811	0.803	0.807	**0.889**
Accuracy	0.725	**0.759**	0.716	0.647	0.733	0.758
Precision	0.805	0.733	**0.829**	0.604	0.798	0.711
Recall	0.593	0.816	0.546	0.855	0.623	**0.893**
F1	0.683	0.772	0.658	0.708	0.699	**0.792**

The best performance is marked in bold, and the second best is underlined.

**Figure 2:**
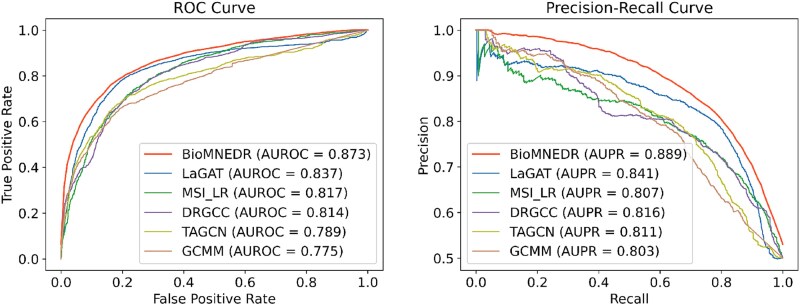
The performance of each method on MSI dataset in terms of ROC and precision–recall (P–R) curves.

BioMNEDR also attains the highest AUROC and AUPR (Fig. 2), demonstrating its strength in balancing classification accuracy with practical utility. The MAX strategy yields slightly lower precision than some baselines, which inherently implies a higher false positive rate. This elevated false positive rate increases the workload of downstream experimental validation in the practical context of drug discovery. Even so, this trade-off is acceptable for real-world applications, as the strategy achieves a higher overall F1-score that reflects an optimal balance between precision and recall. These findings suggest that BioMNEDR is well suited for biomedical applications where comprehensive coverage of potential candidates is essential.

### Ablation study of BioMNEDR components

To evaluate the individual contributions of BioMNEDR components, we conducted an ablation study focusing on network embedding methodologies, prediction algorithms, and integration strategies. The detailed experimental setup is described in the Supplementary Material.

#### Effect of meta-path-based embedding

As presented in Table 5 and Fig. S1, meta-path-based embedding outperforms four network embedding methods (DeepWalk, GraRep, Node2Vec, and SDNE) in AUROC, AUPR, and recall. This advantage is attributed to the capacity of meta-paths to effectively preserve multi-scale biological semantics within heterogeneous MSI networks, while existing methods do not explicitly incorporate biomedical mechanisms.

**Table 5:** Performance comparison of network embedding methods.

Metric	Deepwalk	GraRep	Node2Vec	SDNE	meta-path
AUROC	0.772	0.774	0.809	0.805	**0.873**
AUPR	0.765	0.776	0.807	0.825	**0.889**
Accuracy	0.710	707	0.741	0.736	0.758
Precision	0.714	0.712	**0.748**	0.747	0.711
Recall	0.688	0.696	0.727	0.715	**0.893**
F1	0.701	0.704	0.737	0.731	**0.792**

#### Prediction algorithm comparison

The results in Table 6 and Fig. S2 indicate that the XGBoost-based BioMNEDR demonstrates superior performance compared with alternative prediction algorithms, including Random Forest (RF), Gradient Boosted Decision Trees (GBDT), LightGBM, and CatBoost, as well as the emerging generative transformer-based foundation model TabPFN [35], as evidenced by its highest AUROC, AUPR, F1-score, and recall.

**Table 6:** Performance comparison of prediction methods.

Metric	CatBoost	LightGBM	RF	GBDT	TabPFN [35]	XGBoost
AUROC	0.826	0.840	0.854	0.809	0.809	**0.873**
AUPR	0.839	0.852	0.872	0.820	0.824	**0.889**
Accuracy	0.708	0.735	**0.763**	0.695	0.742	0.758
Precision	0.665	0.695	**0.733**	0.653	0.730	0.711
Recall	0.868	0.862	0.847	0.868	0.789	**0.893**
F1	0.753	0.769	0.786	0.745	0.758	**0.792**

#### Integration strategy evaluation

Comparative experiments, detailed in Table 7 and Fig. S3, reveal that our MAX integration strategy achieves the highest AUROC, AUPR, F1-score, and recall, outperforming alternative approaches. MAX effectively integrates predictions across multiple biological mechanisms without requiring additional semantic modeling. In contrast, alternative methods appear to be less effective at capturing the complex associations arising from multiple biological mechanisms.

**Table 7:** Performance comparison of integration strategies.

Metric	LSTM	LR	MLP	MIN	MEAN	MAX
AUROC	0.815	0.827	0.854	0.870	0.870	**0.873**
AUPR	0.826	0.831	0.879	0.881	0.883	**0.889**
Accuracy	0.735	749	0.773	0.775	**0.795**	0.758
Precision	0.722	0.744	0.742	**0.867**	0.803	0.711
Recall	0.787	0.781	0.866	0.664	0.796	**0.893**
F1	0.753	0.762	0.789	0.752	0.790	**0.792**

Taken together, the observed performance superiority of the BioMNEDR model is a direct consequence of the synergistic integration of meta-path-based embedding, XGBoost classifier, MAX integration strategy. Its leading performance in AUROC, AUPR, and recall demonstrates enhanced classification capabilities and a broader coverage of positive samples, thereby providing an efficient and interpretable computational framework for systematic drug repurposing.

### Case studies of drug repurposing applications

To evaluate the clinical relevance of BioMNEDR, we performed case studies on three complex diseases with substantial unmet therapeutic needs: PD, BC, and AD.

For PD, a neurodegenerative movement disorder caused by dopaminergic neuron loss, most current therapies provide only symptomatic relief. Notably, all top five PD candidate drugs predicted by BioMNEDR are DrugBank-approved, and four of the top 10 are currently under clinical investigation for PD (Table 8 and Table S1). Among these candidates, **rotigotine** [36] was identified as the highest-scoring drug. Its high score is supported by the meta-path “$\mathbf{Dr{-}P{-}Di}$,” correctly reflecting its mechanism as a selective agonist binding to brain dopamine receptors (**P**) to alleviate motor symptoms. Beyond known treatments, the model also identified four promising candidates with distinct PD-relevant mechanisms: flavoxate exerts dopaminergic modulation potential, cyclophosphamide offers dose-dependent immunomodulatory effects, meclizine demonstrates preclinical neuroprotection and blood-brain barrier permeability, and atropine serves as an adjunct for advanced PD-related sialorrhea. These mechanism-aligned predictions highlight the utility of BioMNEDR in identifying biologically plausible candidates for PD while underscoring the necessity of further clinical validation.

**Table 8:** Top 10 candidate drugs for Parkinson’s disease.

Rank	Drug	Original disease	Prediction	Evidence
1	rotigotine	PD	0.992	DrugBank DB05271
2	biperiden	PD, Dyskinetic syndrome	0.992	DrugBank DB00810
3	memantine	PD, AD	0.991	DrugBank DB01043
4	lisuride	PD, Dyskinetic syndrome	0.990	DrugBank DB00589
5	diphenhydramine	PD, Dyskinetic syndrome	0.988	DrugBank DB01075
6	flavoxate	Dysuria, Nocturia	0.987	Ref. [40]
7	cyclophosphamide	Breast Carcinoma, Leukemia	0.975	Ref. [41]
8	citicoline	PD, AD, glaucoma, stroke	0.985	DrugBank DB12153
9	meclizine	vertigo, nausea	0.984	Ref. [42]
10	atropine	asthma, bradycardia	0.984	Ref. [43]

In the context of BC, six of the top ten predicted candidates are approved drugs (Table S2). Leading the list is 5-fluorouracil [37], which exemplifies the efficacy of the meta-path “$\mathbf{Dr{-}P{-}F{-}P{-}Di}$”. A commonly used chemotherapeutic agent, 5-fluorouracil, targets SLC7A11 (**P**)—a key component of the glutamate antiporter—to regulate ferroptosis (**F**), an iron-dependent programmed cell death marked by lipid peroxidation and reactive oxygen species accumulation. This regulation downregulates downstream proteins such as GPX4 (downstream **P**), disrupting redox homeostasis and ultimately suppressing the proliferation, migration, and survival of breast cancer cells.

Regarding AD, BioMNEDR identified multiple high-confidence candidates (Table S3). Physostigmine [17] ranked highest among the predicted candidates, interpreted through the meta-path “$\mathbf{Dr{-}P{-}F{-}P{-}Di}$”: it inhibits acetylcholinesterase (**P**) to enhance cholinergic transmission (**F**), thereby stimulating downstream cholinergic receptors (**P**) to alleviate AD-related cognitive deficits. Another notable candidate is **cromoglicic acid** [38], an agent that has completed Phase III trials, acting via the same meta-path “$\mathbf{Dr{-}P{-}F{-}P{-}Di}$”. It targets functional receptors on microglia (**P**), enhances phagocytosis (**F**) to reverse impaired clearance systems in AD brains, and empowers microglia to engulf pathological A$\beta $ proteins (downstream **P**), thereby alleviating neurotoxicity and synaptic loss.

Comprehensive drug rankings, supporting evidence, and meta-path matching for all case studies, together with the research on the meta-paths underlying drug action mechanisms, are provided in the Supplementary Material (Tables S1–S3).

**Figure 3:**
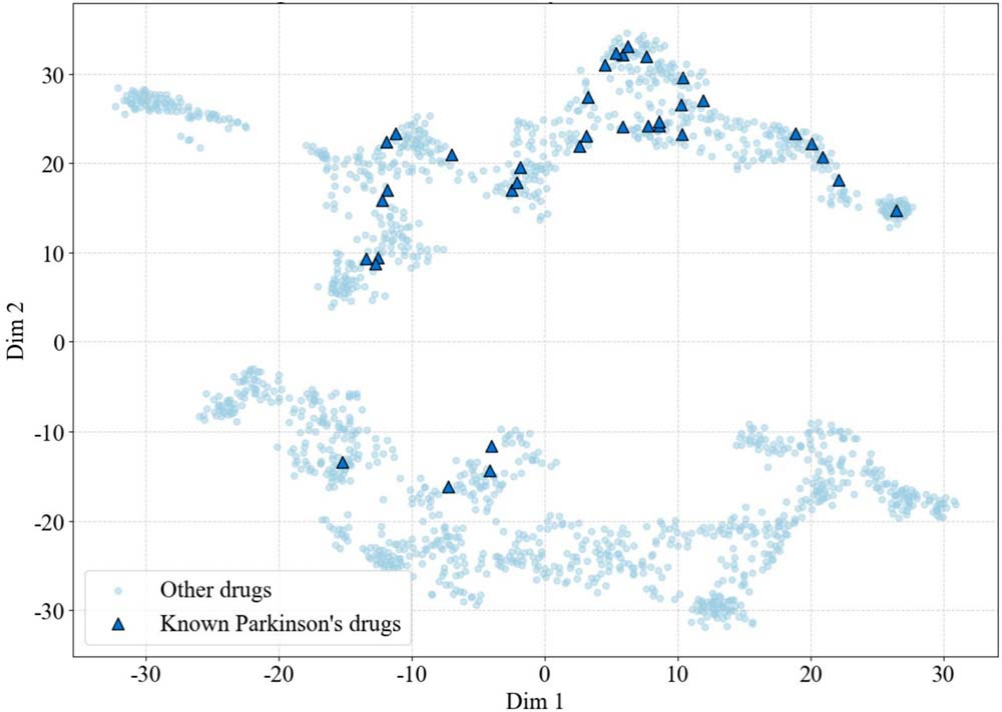
Visualization of drugs in BioMNEDR. Drug entities are embedded into a 2D space via t-SNE [39], utilizing the precomputed drug representation vectors as inputs. Dots represent other drugs, while triangles denote known PD drugs. BioMNEDR effectively captures underlying biomedical relationships (${P_{\mathrm{PD}}}=7.594\times 10^{-34}$).

To visually assess the quality of drug feature representations, we applied t-SNE dimensionality reduction to the complete set of learned drug embeddings. As shown in Fig. 3 and Figs S4 and S5, the visualization revealed clear clustering, with known drugs occupying overlapping regions in the 2D space. Moreover, the cosine distances between embedding vectors of known drugs were significantly smaller than expected under a random distance distribution ($P_{\mathrm{PD}}=7.594\times 10^{-34}$, $P_{\mathrm{BC}}=1.546\times 10^{-9}$, $P_{\mathrm{AD}}=4.383\times 10^{-5}$; two-sample Kolmogorov–Smirnov test). These findings, supported by both statistical evidence and spatial clustering, indicate that BioMNEDR effectively captures underlying biomedical relationships rather than learning spurious associations.

These results demonstrate that BioMNEDR achieves robust repurposing capability for PD, BC, and AD, supported by validation against curated databases and clinical trial evidence. The meta-path-based embedding explicitly models multi-scale biomedical mechanisms by incorporating higher-order relationships among entities, while the ensemble integration strategy alleviates data imbalance and enhances adaptability to real-world applications in drug repurposing.

## Discussion and conclusion

We present **BioMNEDR**, a mechanism-guided network embedding framework for drug repurposing. Unlike most existing black-box network-based methods, BioMNEDR integrates biologically curated meta-paths to capture multi-scale therapeutic mechanisms, thus enhancing both interpretability and predictive performance.

Benchmarking on the MSI network demonstrated that BioMNEDR surpasses state-of-the-art baselines across AUROC, AUPR, recall, and F1-score, with recall gains particularly valuable for minimizing missed therapeutic opportunities. Case studies on PD, BC, and AD further confirmed its clinical relevance, as BioMNEDR successfully rediscovered approved drugs and highlighted candidates under active clinical investigation.

The framework’s strength lies in the integration of three components: meta-path-based embeddings preserving biomedical semantics, an XGBoost classifier optimized for heterogeneous features, and a MAX rule to aggregate predictions across complementary mechanisms. Beyond merely outputting association probabilities, the model further enables inference of drugs’ modes of action via the contributing meta-paths, as demonstrated in our case studies. Together, these elements enable robust and interpretable predictions for drug–disease associations.

Limitations of BioMNEDR include its reliance on the completeness of the MSI network and the requirement for experimental validation to confirm predicted mechanisms. Additionally, the dataset utilized by the method exhibits inherent topological biases, such as the overrepresentation of common diseases in the network and the research tendency to focus on hub-like broad-spectrum drugs. Our future work aims to incorporate temporal, patient-specific, or cell line-specific network data [44, 45] to enhance translational utility, yet this introduces the challenge of leveraging such data without diluting the model’s focus on core therapeutic mechanisms. Taken together, BioMNEDR offers a practical and interpretable computational tool to accelerate systematic drug repurposing.

**Key Points**

- We propose BioMNEDR, a novel mechanism-guided network embedding framework for drug repurposing that integrates a multi-scale heterogeneous biomedical network.
- BioMNEDR designs biologically curated meta-paths (e.g. drug–protein–disease) to explicitly model multi-scale therapeutic mechanisms. These meta-paths facilitate the reconstruction of the mechanistic pathways of repurposed drugs, enhancing biomedical interpretability beyond “black-box” approaches.
- BioMNEDR employs a robust prediction strategy by learning distinct embeddings for each mechanism-specific meta-path and integrating the predictions using an XGBoost classifier with a MAX integration strategy.
- BioMNEDR achieves state-of-the-art performance, outperforming strong baselines across AUROC, AUPR, and notably Recall. Case studies (e.g. Alzheimer’s and Parkinson’s) demonstrate its ability to rediscover approved drugs and prioritize promising clinical candidates.

## Supplementary Material

Supplemental_Material_bbag101

## Data Availability

The data and source code for BioMNEDR can be downloaded from GitHub (https://github.com/boatforoasia/BioMNEDR).
