# Supplementary material for: BioMNEDR: mechanism-guided network embedding for drug repurposing
Source: Brief Bioinform. 2026 Mar 9;27(2):bbag101. doi: 10.1093/bib/bbag101 (PMC12971018; doi:10.1093/bib/bbag101)
Supplement: Supplemental_Material_bbag101 [file supplemental_material_bbag101.pdf]

Supplemental Material for
BioMNEDR: Mechanism-Guided Network Embedding for Drug Repurposing

Yizhou Zeng[1,†], Lei Wang[2,†], Xueming Liu[2,*]

[1]School of Future Technology,
Huazhong University of Science and Technology,
Luoyu Road, Wuhan 430074, China

[2]School of Artificial Intelligence and Automation,
State Key Laboratory of Digital Manufacturing Equipments and Technology,
Institute of Medical Equipment Science and Engineering,
Huazhong University of Science and Technology,
Wuhan, China

∗ xm_liu@hust.edu.cn

† These authors contributed equally to this work.

1. FIGURES AND TABLES

A. Supplementary Figures

The effects of three key modifications were evaluated through ablation experiments: the graph embedding method (Figure S1), the prediction method (Figure S2), and the integration strategy (Figure S3), with corresponding AUROC and AUPR curves presented in each figure.

Figure S4 shows the visualization of drugs. The drug entities were embedded into a two-dimensional space via t-SNE , using the precomputed drug representation vectors as input. Light blue dots ('Other drugs') represent the broader drug space, while yellow stars denote known Alzheimer's disease drugs.

Figure S5 shows the visualization of drugs. The drug entities were embedded into a two-dimensional space via t-SNE, using the precomputed drug representation vectors as input. Light blue dots ('Other drugs') represent the broader drug space, while pink dots denote known breast cancer drugs.

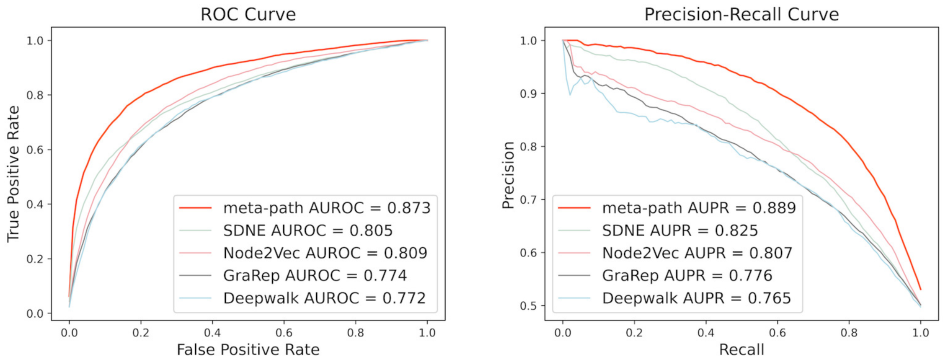

Fig. S1. The performance of each graph embedding method in terms of ROC and precision–recall (P–R) curves.

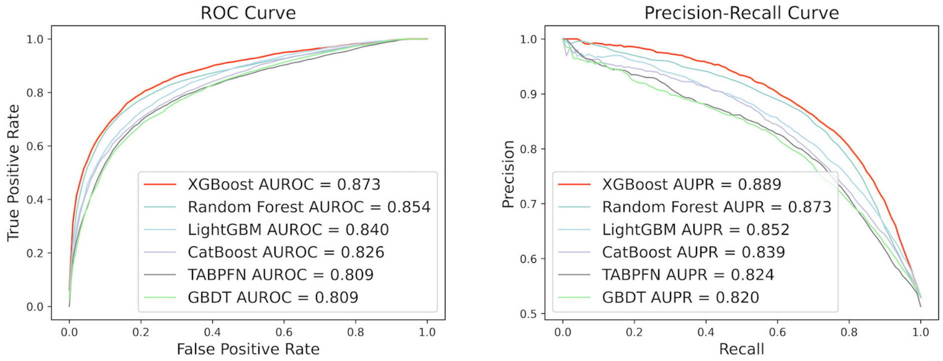

Fig. S2. The performance of each prediction method in terms of ROC and precision–recall (P–R) curves.

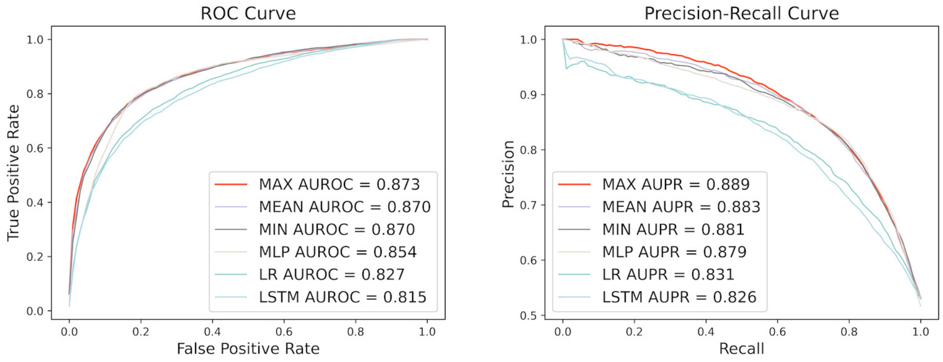

Fig. S3. The performance of each integration strategy in terms of ROC and precision–recall (P–R) curves.

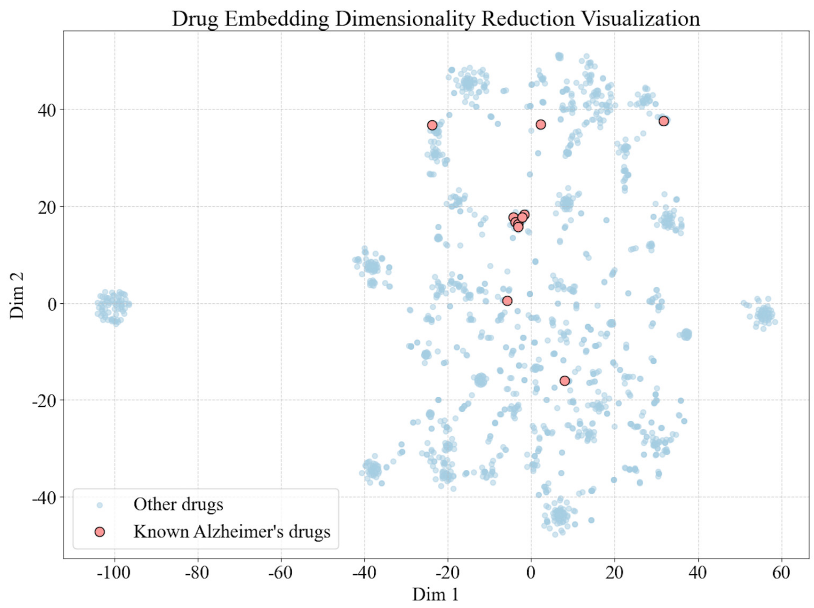

Fig. S4. Visualization of known Alzheimer's disease drugs and other drugs.

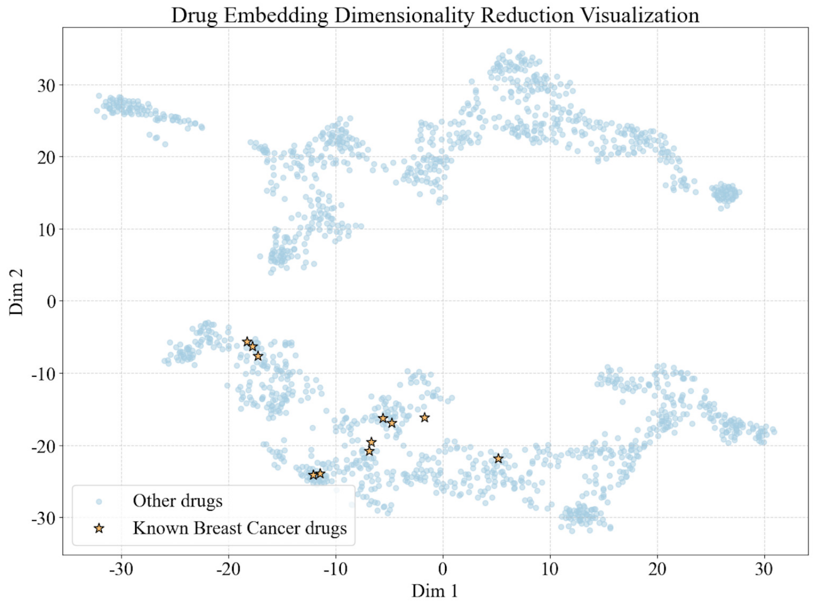

Fig. S5. Visualization of known breast cancer drugs and other drugs.

B. Supplementary Tables

Table S1, S2 and S3 enumerate the top 30 predicted drugs for Parkinson's disease, breast cancer, and Alzheimer's disease, respectively. Herein, the symbols represent distinct meta-paths as follows: (a) denotes $Dr - P - Di$; (b) denotes $Dr - P - P - Di$; (c) denotes $Dr - P - F - P - Di$; (d) denotes $Dr - P - F - F - P - Di$.

Parkinson's disease, a movement disorder caused by dopaminergic neuron degeneration, urgently requires therapies that transcend symptomatic management. The top five drugs ranked by probability are all drugs already existing in the database.

Additionally, four new drugs under clinical investigation have been identified. Remarkably, meclizine, a widely used antihistamine, has recently revealed neuroprotective properties, particularly demonstrating therapeutic potential in PD cellular models[1].

For breast cancer treatment, our study revealed that six of the top 10 highest-scoring potential drug candidates have been validated as effective in DrugBank[2], including 5-fluorouracil, paclitaxel, ixabepilone, doxorubicin, cyclophosphamide, and everolimus while the remaining four are supported by preclinical evidence from existing studies. Specifically, the model successfully identified standard chemotherapeutic agents (e.g., 5-fluorouracil, ranked 1st) and innovative mechanism-driven candidates. For instance, norfloxacin (ranked 6th), an antibiotic originally indicated for prostatitis, was highly ranked. Experimental studies[3] confirm that certain norfloxacin derivatives exhibit significant anti-proliferative activity against breast cancer cell lines, aligning with model predictions. Similarly, etoposide (ranked 10th), approved for lung cancer, demonstrates clinical relevance in metastatic breast cancer retreatment strategies[4], further validating the biological plausibility of predictions.

Alzheimer's disease, a neurodegenerative disorder characterized by β-amyloid plaques and tau protein tangles, currently lacks disease-modifying therapies that can decelerate pathological progression and improve patients' quality of life beyond symptomatic relief. Notably, in Table 8, a Phase 3-completed drug[2], cromoglicic acid stands out. It underscores the model's capacity to identify high-viability candidates with established clinical relevance. This outcome not only validates the algorithm's biological fidelity but also highlights its utility in accelerating drug repurposing pipelines for complex diseases.

Table S1. Top 30 candidate drugs for Parkinson's disease

Rank	Drug	Original Disease	Prediction	Evidence (Meta-path)
1	rotigotine	PD	0.992	DrugBank DB05271 (a)
2	biperiden	PD, Dyskinetic Syndrome	0.992	DrugBank DB00810 (a)
3	memantine	PD, AD	0.991	DrugBank DB01043 (a)
4	lisuride	PD, Dyskinetic Syndrome	0.990	DrugBank DB00589 (a)
5	diphenhydramine	PD, Dyskinetic Syndrome	0.988	DrugBank DB01075 (a)
6	flavoxate	Dysuria, Nocturia	0.987	[5] (c)
7	cyclophosphamide	Breast Carcinoma, Leukemia	0.975	[6] (c)
8	citicoline	PD, AD, glaucoma, stroke	0.985	DrugBank DB12153 (c)
9	meclizine	vertigo, nausea	0.984	[7] (c)
10	atropine	asthma, bradycardia	0.984	[8] (a)
11	profenamine	PD	0.984	DrugBank DB00392 (a)
12	methylprednisolone	asthma	0.984	[9] (c)
13	nemonapride	schizophrenia	0.984	/
14	dopamine	PD	0.984	DrugBank DB00988 (c)
15	droperidol	nausea	0.983	/
16	selegiline	PD	0.983	DrugBank DB01037 (d)
17	levomepromazine	schizophrenia	0.983	/
18	caramiphen	PD	0.983	/
19	hydroxyurea	melanoma	0.982	/
20	risperidone	schizophrenia	0.982	[10] (a)
21	benztropine-mesylate	PD	0.982	/
22	Pergolide	PD	0.981	DrugBank DB01186 (a)
23	pilocarpine	Xerostomia	0.981	/
24	ropinirole	PD	0.981	DrugBank DB00268 (a)
25	magnesium carbonate	heartburn	0.981	[11] (c)
26	felbamate	Liver Failure	0.980	/
27	prochlorperazine	ausea	0.980	/
28	pargyline	Hypertensive disease	0.980	/
29	bromperidol	schizophrenia	0.979	/
30	norepinephrine	asthma	0.978	/

Table S2. Top 30 candidate drugs for Breast Cancer

Rank	Drug	Original Disease	Prediction	Evidence (Meta-path)
1	5-fluorouracil	Breast Carcinoma	0.986	Drug Bank DB00544 (c)
2	paclitaxel	Breast Carcinoma	0.983	Drug Bank DB01229 (a)
3	Peginterferon alfa-2b	Hemangioma	0.980	[12] (a)
4	ixabepilone	Breast Carcinoma, RCC	0.978	DrugBank DB04845 (c)
5	doxorubicin	Breast Carcinoma, NHL	0.977	DrugBank DB00997 (c)
6	norfloxacin	prostatitis	0.976	[3] (a)
7	cyclophosphamide	Breast Carcinoma, CLL	0.975	DrugBank DB00531 (a)
8	irinotecan	Lymphoma	0.971	[13] (c)
9	everolimus	Breast Carcinoma	0.971	DrugBank DB01590 (b)
10	etoposide	Brain Neoplasms	0.970	[4] (c)
11	docetaxel	melanoma	0.968	DrugBank DB01248 (c)
12	vinorelbine	Mammary Neoplasms	0.965	[14] (a)
13	methotrexate	Leukemia	0.964	[15] (b)
14	dactinomycin	melanoma	0.963	[16] (a)
15	tretinoin	Leukemia	0.962	[17] (b)
16	bleomycin	Lymphoma	0.961	[18] (d)
17	Nandrolone phenpropionate	Breast Carcinoma	0.961	DrugBank DB00984 (c)
18	epirubicin	Breast Carcinoma	0.961	DrugBank DB00445 (c)
19	gemcitabine	Adenocarcinoma Pancreas	0.959	/
20	Drostanolone	rosacea	0.959	/
21	moxifloxacin	peritonitis	0.959	/
22	vinblastine	Mammary Neoplasms	0.958	/
23	levofloxacin	sinusitis	0.958	[19] (a)
24	Interferon gamma-1b	Hemangioma	0.958	/
25	cisplatin	neuroblastoma	0.957	[20] (c)
26	fleroxacin	diarrhea	0.956	/
27	Nandrolone decanoate	Growth Failure	0.952	DrugBank DB08804 (a)
28	temsirolimus	Lymphoma	0.951	/
29	ofloxacin	Cystitis	0.951	/
30	topotecan	Ovarian Neoplasm	0.949	[21] (c)

Table S3. Top 30 candidate drugs for Alzheimer's disease

Rank	Drug	Original Disease	Prediction	Evidence (Meta-path)
1	physostigmine	AD, glaucoma	0.985	DrugBank DB00981 (c)
2	prednisone	Drug Allergy, NHL, CTCL	0.984	[22]
3	rivastigmine	PD, AD	0.981	DrugBank DB00989 (c)
4	tacrine	AD	0.977	DrugBank DB00382 (a)
5	ranitidine	Duodenal Ulcer	0.974	[23]
6	edrophonium	myasthenia gravis	0.974	-
7	pralidoxime	myasthenia gravis	0.967	-
8	Valproic Acid	AD, seizures	0.967	DrugBank DB00313 (c)
9	Hydrocortisone	Cerebral Edema	0.961	[24]
10	cromoglicic-acid	asthma, psoriasis	0.949	[25]; Phase III completed (DrugBank DB00377) (c)
11	cortisone-acetate	Pemphigus, asthma	0.945	/
12	triamcinolone	Keloid	0.944	/
13	citicoline	PD, AD	0942	DrugBank DB12153 (d)
14	NADH	AD, PD	0.937	DrugBank DB00157 (d)
15	memantine	AD, PD	0.933	DrugBank DB01043 (a)
16	papaverine	colitis	0.932	/
17	DB00033	skin ulcer	0.927	/
18	Choline	sleeplessness	0.926	/
19	cinacalcet	hypercalcemia	0.926	/
20	pentoxifylline	stroke	0.926	/
21	Peginterferon alfa-2a	melanoma	0.925	/
22	metoclopramide	nausea	0.925	/
23	phenylephrine	fever	0.925	[26]
24	streptozotocin	Hodgkin's lymphoma	0.922	/
25	selegiline	AD, PD	0.920	/
26	elbamate	Liver Failure	0.920	/
27	carmustine	ependymoma	0.917	/
28	donepezil	AD	0.913	/
29	cyclophosphamide	Lymphoma	0.911	/
30	magnesium carbonate	heartburn	0.908	/

2. EQUATION

We have a set of weighted random walk sequences $\mathcal{W} = \{v_1, v_2, ..., v_M\}$ guided by meta-path $\mathcal{P}$. The mathematical formulations for deriving node embeddings are presented below.

A. Heterogeneous Skip-Gram Objective

For each node $v \in \mathcal{W}$, maximize the log-probability of observing its heterogeneous neighborhood $N_t(v)$. The objective function is:

$$\begin{aligned} \mathcal{O}(\mathbf{X}) = \sum_{v \in W} \sum_{t \in T_v} \sum_{c_t \in N_t(v)} & [\log \sigma(\mathbf{X}_{c_t} \cdot \mathbf{X}_v) \\ & + \sum_{m=1}^{M} \mathbb{E}_{u_t^m \sim P_t(u)} \log \sigma(-\mathbf{X}_{u_t^m} \cdot \mathbf{X}_v)] \end{aligned} \tag{S1}$$

where $\mathbf{X}_v$ represents the embedding vector of node v, $\sigma(x) = \frac{1}{1+e^{-x}}$ represents sigmoid function, $N_t(v)$ represents nodes of type t in the context window of v, $P_t(u)$ represents sampling distribution for negative nodes of type t.

B. Conditional Probability with Type-Specific Softmax

For a taget node v and a context node c_t of type t, the probability is:

$$p(c_t | v; \theta) = \frac{\exp(\mathbf{X}_{c_t} \cdot \mathbf{X}_v)}{\sum_{u \in V_t} \exp(\mathbf{X}_u \cdot \mathbf{X}_v)} \tag{S2}$$

where V_t is the set of nodes of type t (e.g., V_D: all drug nodes).

C. Negative Sampling for Heterogeneous Types

For each positive pair (v, c_t), sample M negative nodes $\{u_t^1, u_t^2, ..., u_t^M\}$ only from nodes of type t. For example: if c_t is a protein (P), sample negatives from V_P.

D. Gradient Updates

Update embeddings using stochastic gradient descent (SGD). For each (v, c_t):
Positive gradient: $\frac{\partial o}{\partial \mathbf{X}_v} \propto \sigma(\mathbf{X}_{c_t} \cdot \mathbf{X}_v) \cdot \mathbf{X}_{c_t}$
Negative gradient: $\frac{\partial \boldsymbol{v}}{\partial \mathbf{x}_v} \propto \sum_{m=1}^{M} \sigma(-\mathbf{X}_{u_t}{}^m \cdot \mathbf{X}_v) \cdot \mathbf{X}_{u_t}{}^m$

3. ALGORITHM

In this work, we set the parameters of the weighted random walks to: (1) The number of walks per node w: 1000; (2) The walk length l:100. We set the parameters of the heterogeneous skip-gram model to: (1) The vector dimension d: 128; (2) The neighborhood size k: 7; (3) The size of negative samples: 5. We set the parameters of the XGBoost model to: (1) The number of booster rounds: 250; (2) The maximum depth of below: 10.

To systematically evaluate model performance, a stratified five-fold cross-validation protocol was implemented, ensuring consistent preservation of therapeutic association class distributions across all data partitions.

4. EXPERIMENTAL SETUP

We systematically conducted ablation experiments to evaluate the contributions of three core components: graph embedding methods, prediction methods, and integration strategies.

Firstly, we substituted meta-path-based embedding (meta-path) with four alternative graph representation learning methods:

- **DeepWalk** [27] learns node embeddings by treating random walks on a graph as sentences and applying word2vec to learn node representations.
- **GraRep** [28] learns node embeddings by capturing high-order graph information through SVD on the graph's adjacency matrix raised to different powers.
- **Node2Vec** [29] learns node embeddings by using biased random walks to explore the graph in a way that balances breadth-first and depth-first search strategies.
- **SDNE** [30] learns node embeddings by using a deep autoencoder to simultaneously capture the first-order and second-order proximity between nodes in a graph.

Figure S1 demonstrates that meta-path outperforms other graph embedding approaches in ROC and precision-recall (P-R) curves. Based on the previous analysis of the meta-path method, this superiority stems from its semantic modeling capability for heterogeneous graphs. Traditional methods often suffer from semantic noise interference in protein-dominated MSI networks due to their neglect of node type heterogeneity, whereas meta-path preserves multi-scale biological semantics through meta-path-guided embedding learning.

Next, in comparative experiments on prediction methods the XGBoost-based BioMNEDR model achieves superior performance across ROC and P-R curves, as shown in Figure S2. The other prediction methods are listed as follows.

- **Random Forest (RF)** [31] is an ensemble learning method that constructs multiple decision trees and averages their predictions to improve accuracy and prevent overfitting.
- **Gradient Boosted Decision Trees (GBDT)** [32] GBDT is an ensemble learning method that sequentially builds decision trees, where each tree corrects the errors of its predecessors by fitting to the negative gradient of the loss function.
- **LightGBM** [33] is a gradient boosting framework that uses tree-based learning algorithms with optimizations like gradient-based one-side sampling (GOSS) and exclusive feature bundling (EFB) for faster training and higher efficiency.
- **CatBoost** [34] is a gradient boosting algorithm that handles categorical features natively and uses ordered boosting to reduce gradient bias and improve accuracy.
- **TabPFN** [35] is a generative transformer-based foundation model for tabular data that can perform few-shot learning without requiring extensive training on new datasets.

The gradient-boosted tree ensemble in XGBoost effectively captures complex nonlinear relationships in drug-disease associations through second-order gradient optimization, while its L1 and L2 regularization mechanisms mitigate overfitting caused by high-dimensional sparse embedding features.

Finally, we replace BioMNEDR's MAX integration strategy ("association is confirmed if predicted by any meta-path") with alternative approaches, including:

- **Long Short-Term Memory (LSTM)** [36] is a type of recurrent neural network (RNN) architecture designed to handle the vanishing gradient problem and capture long-range dependencies in sequential data.
- **Logistic Regression (LR)** [37] is a linear model used for binary classification that predicts the probability of a binary outcome using a logistic function.
- **Multilayer Perceptron (MLP)** [38] is a type of feedforward artificial neural network that consists of multiple layers of nodes, each fully connected to the next, used for both classification and regression tasks.
- **MEAN** is a dynamic ensemble strategy that combines the predictions of multiple models by taking the average of their outputs.
- **MIN** is a dynamic ensemble strategy that selects the minimum value among the predictions of multiple models as the final prediction.

As evidenced by AUROC and AUPR curves in Figure S3, BioMNEDR exhibits superior overall performance. This likely occurs because BioMNEDR directly leverages first-layer outputs without requiring additional modeling of heterogeneous graph semantics. In contrast, methods relying on LSTM or MLP struggle to cover multi-mechanism associations due to their fixed-weight or single-perspective limitations, while BioMNEDR reduces missed detection risks through meta-path complementarity. Although its precision slightly trails the MEAN strategy, the clinical value of high recall in drug repositioning outweighs the precision compromise.

5. EVALUATION METRICS

Comparative experiments were conducted on the same dataset with comprehensive evaluation across six metrics:

- **Area Under the Receiver Operating Characteristic Curve (AUROC)** measures the ability of a classifier to distinguish between classes, representing the probability that a randomly chosen positive instance is ranked higher than a randomly chosen negative instance.
- **Area Under the Precision-Recall Curve (AUPR)** summarizes the precision-recall curve, providing a measure of a classifier's performance, particularly useful when dealing with imbalanced datasets.
- **Accuracy** represents the proportion of correctly classified instances out of the total number of instances.

- **Precision** measures the proportion of true positive predictions out of all positive predictions, indicating how well the model avoids false positives.
- **Recall** measures the proportion of true positive predictions out of all actual positive instances, indicating how well the model avoids false negatives.
- **F1-score** is the harmonic mean of precision and recall, providing a balanced measure of a classifier's performance.

Recall is prioritized to mitigate the irreversible scientific costs of false negatives, which represent missed therapeutic candidates, ensuring comprehensive identification of potential drug-disease associations. This strategy aligns with principles of clinical diagnostics and drug discovery [39].

REFERENCES

1. C. T. Hong, K.-Y. Chau, and A. H. Schapira, "Meclizine-induced enhanced glycolysis is neuroprotective in parkinson disease cell models," Sci. reports **6**, 25344 (2016).
2. C. Knox, M. Wilson, C. M. Klinger, *et al.*, "Drugbank 6.0: the drugbank knowledgebase for 2024," Nucleic acids research **52**, D1265–D1275 (2024).
3. A. N. Fallica, C. Barbaraci, E. Amata, *et al.*, "Nitric oxide photo-donor hybrids of ciprofloxacin and norfloxacin: a shift in activity from antimicrobial to anticancer agents," J. Medicinal Chem. **64**, 11597–11613 (2021).
4. G. Giannone, A. Milani, E. Ghisoni, *et al.*, "Oral etoposide in heavily pre-treated metastatic breast cancer: A retrospective series," The Breast **38**, 160–164 (2018).
5. Y. Açar, D. Ağagündüz, P. De Cicco, and R. Capasso, "Flavonoids: Their putative neurologic roles, epigenetic changes, and gut microbiota alterations in parkinson's disease," Biomed. & Pharmacother. **168**, 115788 (2023).
6. K. M. Ibrahim, S. F. Darwish, E. M. Mantawy, and E. El-Demerdash, "Molecular mechanisms underlying cyclophosphamide-induced cognitive impairment and strategies for neuroprotection in preclinical models," Mol. Cell. Biochem. **479**, 1873–1893 (2024).
7. R. E. Mostafa and G. F. Asaad, "Meclizine moderates lipopolysaccharide-induced neuroinflammation in mice through the regulation of akt/nf-$\kappa\beta$/erk/jnk signaling pathway," Metab. Brain Dis. **38**, 2797–2806 (2023).
8. P. Kempster and A. Ma, "Parkinson's disease, dopaminergic drugs and the plant world," Front. pharmacology **13**, 970714 (2022).
9. Y. Sato, T. Asoh, N. Metoki, and K. Satoh, "Efficacy of methylprednisolone pulse therapy on neuroleptic malignant syndrome in parkinson's disease," J. Neurol. Neurosurg. & Psychiatry **74**, 574–576 (2003).
10. E. Mohr, T. Mendis, K. Hildebrand, and P. P. De Deyn, "Risperidone in the treatment of dopamine-induced psychosis in parkinson's disease: An open pilot trial," Mov. Disord. Off. J. Mov. Disord. Soc. **15**, 1230–1237 (2000).
11. K. Oyanagi and T. Hashimoto, "Magnesium in parkinson's disease: an update in clinical and basic aspects," (2018).

12. P. Mishra, B. Nayak, and R. Dey, "Pegylation in anti-cancer therapy: An overview," Asian journal pharmaceutical sciences **11**, 337–348 (2016).
13. H. Lan, Y. Li, and C.-Y. Lin, "Irinotecan as a palliative therapy for metastatic breast cancer patients after previous chemotherapy," Asian Pac. J. Cancer Prev. **15**, 10745–10748 (2015).
14. Y.-C. Xu, H.-X. Wang, L. Tang, *et al.*, "A systematic review of vinorelbine for the treatment of breast cancer," The breast journal **19**, 180–188 (2013).
15. J. Raut, O. Sarkar, T. Das, *et al.*, "Efficient delivery of methotrexate to mda-mb-231 breast cancer cells by a ph-responsive zno nanocarrier," Sci. Reports **13**, 21899 (2023).
16. H. Yang, S. Li, W. Li, *et al.*, "Actinomycin d synergizes with doxorubicin in triple-negative breast cancer by inducing p53-dependent cell apoptosis," Carcinogenesis **45**, 262–273 (2024).
17. M. Wu, M. Kim, Y. Chen, *et al.*, "Retinoic acid directs breast cancer cell state changes through regulation of tet2-pkcζ pathway," Oncogene **36**, 3193–3206 (2017).
18. M. Kullberg, K. Mann, and T. J. Anchordoquy, "Targeting her-2+ breast cancer cells with bleomycin immunoliposomes linked to llo," Mol. pharmaceutics **9**, 2000–2008 (2012).
19. M. Yu, R. Li, and J. Zhang, "Repositioning of antibiotic levofloxacin as a mitochondrial biogenesis inhibitor to target breast cancer," Biochem. biophysical research communications **471**, 639–645 (2016).
20. H. Wang, S. Guo, S.-J. Kim, *et al.*, "Cisplatin prevents breast cancer metastasis through blocking early emt and retards cancer growth together with paclitaxel," Theranostics **11**, 2442 (2021).
21. K. A. Jaeckle, J. G. Dixon, S. K. Anderson, *et al.*, "Intra-csf topotecan in treatment of breast cancer patients with leptomeningeal metastases," Cancer medicine **9**, 7935–7942 (2020).
22. P. S. Aisen, K. Davis, J. Berg, *et al.*, "A randomized controlled trial of prednisone in alzheimer's disease," Neurology **54**, 588–588 (2000).
23. J. Gao, N. Midde, J. Zhu, *et al.*, "Synthesis and biological evaluation of ranitidine analogs as multiple-target-directed cognitive enhancers for the treatment of alzheimer's disease," Bioorganic & medicinal chemistry letters **26**, 5573–5579 (2016).
24. J. Li, L. Chen, S. Liu, *et al.*, "Hydrocortisone mitigates alzheimer's-related cognitive decline through modulating oxidative stress and neuroinflammation," Cells **12**, 2348 (2023).
25. C. Zhang, A. Griciuc, E. Hudry, *et al.*, "Cromolyn reduces levels of the alzheimer's disease-associated amyloid β-protein by promoting microglial phagocytosis," Sci. reports **8**, 1144 (2018).
26. M. M. Elseweidy, M. Mahrous, S. I. Ali, *et al.*, "Pentoxifylline as add-on treatment to donepezil in copper sulphate-induced alzheimer's disease-like neurodegeneration in rats," Neurotox. research **41**, 546–558 (2023).
27. B. Perozzi, R. Al-Rfou, and S. Skiena, "Deepwalk: Online learning of social rep-

resentations," in *Proceedings of the 20th ACM SIGKDD international conference on Knowledge discovery and data mining,* (2014), pp. 701–710.

28. S. Cao, W. Lu, and Q. Xu, "Grarep: Learning graph representations with global structural information," in *Proceedings of the 24th ACM international on conference on information and knowledge management,* (2015), pp. 891–900.
29. A. Grover and J. Leskovec, "node2vec: Scalable feature learning for networks," in *Proceedings of the 22nd ACM SIGKDD international conference on Knowledge discovery and data mining,* (2016), pp. 855–864.
30. D. Wang, P. Cui, and W. Zhu, "Structural deep network embedding," in *Proceedings of the 22nd ACM SIGKDD international conference on Knowledge discovery and data mining,* (2016), pp. 1225–1234.
31. L. Breiman, "Random forests," Mach. learning **45**, 5–32 (2001).
32. S. Si, H. Zhang, S. S. Keerthi, *et al.*, "Gradient boosted decision trees for high dimensional sparse output," in *International conference on machine learning,* (PMLR, 2017), pp. 3182–3190.
33. G. Ke, Q. Meng, T. Finley, *et al.*, "Lightgbm: A highly efficient gradient boosting decision tree," Adv. neural information processing systems **30** (2017).
34. L. Prokhorenkova, G. Gusev, A. Vorobev, *et al.*, "Catboost: unbiased boosting with categorical features," Adv. neural information processing systems **31** (2018).
35. N. Hollmann, S. Müller, L. Purucker, *et al.*, "Accurate predictions on small data with a tabular foundation model," Nature **637**, 319–326 (2025).
36. S. Hochreiter and J. Schmidhuber, "Long short-term memory," Neural computation **9**, 1735–1780 (1997).
37. D. W. Hosmer Jr, S. Lemeshow, and R. X. Sturdivant, *Applied logistic regression* (John Wiley & Sons, 2013).
38. A. Pinkus, "Approximation theory of the mlp model in neural networks," Acta numerica **8**, 143–195 (1999).
39. F. Gentile, J. C. Yaacoub, J. Gleave, *et al.*, "Artificial intelligence–enabled virtual screening of ultra-large chemical libraries with deep docking," Nat. Protoc. **17**, 672–697 (2022).
